# Mannitol as a Critical Excipient in Spray-Dried Chitosan Microspheres for Nasal Donepezil Delivery: Insights from Integrated Biomimetic Models

**DOI:** 10.3390/pharmaceutics18081023

**Published:** 2026-08-18

**Authors:** Mirna Perkušić, Laura Nižić Nodilo, Mario Jug, Cvijeta Jakobušić Brala, Regina Scherließ, Anita Hafner

**Affiliations:** 1 Faculty of Pharmacy and Biochemistry, University of Zagreb, 10000 Zagreb, Croatia; mirna.perkusic@pharma.unizg.hr (M.P.); laura.nizic@pharma.unizg.hr (L.N.N.); mario.jug@pharma.unizg.hr (M.J.); cvijeta.jakobusic@pharma.unizg.hr (C.J.B.); 2 Department of Pharmaceutics and Biopharmaceutics, Faculty of Mathematics and Natural Sciences, Kiel University, 24143 Kiel, Germany; 3 Priority Research Area Kiel Nano, Surface and Interface Sciences (KiNSIS), Kiel University, 24143 Kiel, Germany

**Keywords:** nose-to-brain, spray-drying, mannitol, sensory effect, nasal powders, slug mucosal irritation assay, ultrasonic nozzle, nasal biomimetic method

## Abstract

**Background/Objectives:** The aim of this study was to develop and apply a novel integrated approach for predicting local mucosal tolerability of spray-dried chitosan/mannitol microspheres previously developed for nose-to-brain donepezil delivery. **Methods:** Microspheres were prepared by ultrasonic spray-drying, and process reproducibility was evaluated based on particle size distribution, entrapment efficiency, and process yield across independent batches. A lactose-based formulation served as a comparative control. A novel biomimetic model was developed to investigate water evaporation under simulated nasal conditions, enabling prediction of formulation dehydration and crust-like layer formation on the nasal mucosa during nasal residence time. Donepezil-loaded chitosan microspheres and their physical mixture with mannitol were used as controls. Analyses were complemented by solid-state and rheological characterization to elucidate the effects of formulation composition and processing on the observed behavior. Irritation potential was further assessed using the established slug mucosal irritation (SMI) assay. **Results:** Reproducible microsphere size distribution (Dv10 11.5 ± 1.1 µm, RSD 9.6%; Dv50 28.4 ± 3.9 µm, RSD 13.7; Dv90 61.3 ± 8.4 µm, RSD 8.4%), entrapment efficiency (99.6 ± 1.8%, RSD 1.8%) and process yield (40.9 ± 5.5%, RSD 13.3%) confirmed the robustness of the ultrasonic spray-drying. Replacing mannitol with lactose failed to achieve the desired particle size distribution, highlighting the key role of mannitol under the investigated processing conditions. The biomimetic model coupled with rheological studies demonstrated that chitosan-based gels formed by microsphere swelling in simulated nasal fluid, maintain viscosity, resist dehydration, and undergo rehydration. Additionally, mannitol enhanced water retention and reduced evaporation without increasing occlusivity or the risk of mucosal dehydration. Furthermore, powders containing mannitol exhibited a lower irritation potential in the SMI assay compared to chitosan microspheres alone. **Conclusions:** Mannitol is a critical determinant of the performance of donepezil-loaded chitosan-based microspheres, contributing to the desired particle size distribution, process reproducibility, favorable hydration and improved mucosal tolerability, thereby supporting the suitability of this platform for nasal donepezil delivery.

## 1. Introduction

Development of nose-to-brain delivery systems constitutes a rapidly evolving research field addressing key limitations in drug solubility, permeability, stability, and nasal residence time, to better exploit the potential of the nasal route [1]. Among advanced delivery platforms, systems that are simple to manufacture and readily scalable, such as swellable powder formulations, have attracted considerable attention. These systems can incorporate mucoadhesive polymers [2], permeation enhancers [3], and/or nanocarriers [4], thereby optimizing nasal residence time as well as drug release and absorption profiles.

Compared with liquid formulations, nasal powders offer several important advantages, including improved stability without the need for preservatives, prolonged retention at the nasal mucosa, higher local drug concentrations, and reduced dependence on patient coordination during administration [5,6,7]. In addition, powder formulations minimize formulation runoff into the throat, thereby reducing discomfort and unpleasant aftertaste. The potential of nasal powders for nose-to-brain drug delivery has been demonstrated in animal studies [3,8,9,10,11]. Moreover, recent reports indicate more efficient olfactory deposition of nasal powders [2,12,13] compared to results reported for nebulized liquid formulations [14,15].

Spray-drying, as a single-step, rapid, and scalable technique, has become a key method for the preparation of nasal powders [7]. It enables precise control over microparticle size and morphology, which is critical for nasal administration. By fine-tuning formulation and process parameters, the radial distribution of components during drying can be controlled, thereby influencing particle size, density, and morphology through changes in evaporation rate and heat-mass transfer [16]. Consequently, geometric and aerodynamic diameter of spray-dried particles can be tailored by selecting appropriate excipients and optimizing process parameters, including feed solid content, inlet temperature, atomizing capacity, and feed rate [7,17,18].

**Figure 1:**
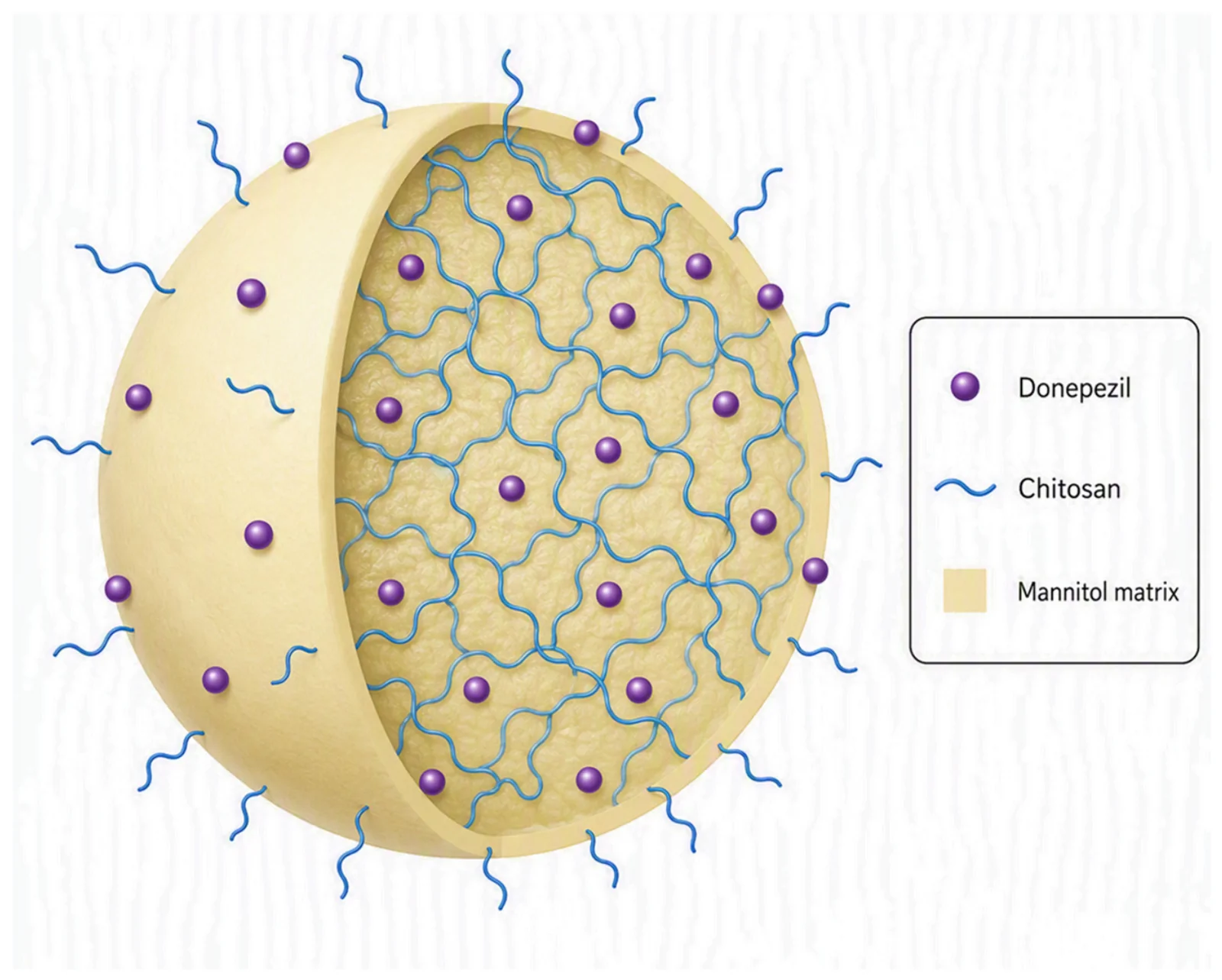
Schematic representation of donepezil-loaded chitosan/mannitol microspheres derived from the characterization results obtained in the previous study [13]. The figure is created with Illustrae.co (UK).

According to current regulatory recommendations for localized nasal delivery, the majority of aerosolized particles should exceed 10 μm in size to ensure deposition within the nasal cavity [19]. While geometric particle size describes the physical dimensions of particles, aerodynamic diameter is a more relevant predictor of the site and efficiency of particle deposition in the respiratory tract because it incorporates the effects of particle density and shape [20,21]. Ultimately, assessment of nasal deposition pattern using anatomically relevant nasal cast models provides direct and physiologically relevant aerodynamic assessment of nasal powder formulations, as it integrates the combined effects of particle size, density, morphology, delivery device, and administration conditions on regional deposition [22]. In our previous work, we developed a free-flowing, spray-dried platform of donepezil-loaded chitosan/mannitol microspheres with particle size optimized for nasal delivery. This formulation demonstrated promising in vitro performance, indicating its potential for efficient brain-targeted delivery of donepezil, as evidenced by high olfactory deposition (65.5%) in a 3D-printed nasal cast, along with favorable swelling, mucoadhesion, drug release, and permeation-enhancing properties [13]. The schematic representation of the microspheres is presented in Figure 1.

Despite these encouraging results, another critical aspect of nasal powder delivery warrants consideration at this stage of formulation development, namely, the prediction of local mucosal tolerability. Reliable biomimetic models are essential for evaluating such effects. It is well recognized that deposited powder particles may induce irritation, sneezing, itching, or pain [23]. However, to date, very few studies have addressed this issue.

The slug mucosal irritation (SMI) assay, developed by Lenoir et al., represents a promising approach for predicting sensory effects on human nasal mucosa, based on the correlation between mucus production in slugs and the incidence of stinging, itching, and burning sensations in humans [24]. This method has been applied to assess the irritancy of nasal liquid formulations [25,26] and nasal powder excipients [23], enabling highly reproducible differentiation between the samples. SMI assay aligns with the principles of the 3Rs (Replacement, Reduction, and Refinement) by supporting the development of alternative testing methods and the use of lower organisms, such as invertebrates, instead of vertebrate animals [24,26]. To the best of our knowledge, the SMI assay is currently the only approach capable of screening sensory effects on the nasal mucosa without the use of vertebrate animal or human studies.

Furthermore, in the case of swellable particles that form a gel layer at the site of deposition, potential formulation dehydration and crust-like layer formation during nasal residence time may lead to discomfort and reduced patient adherence [27]. Nevertheless, as far as we are aware, no studies have investigated the potential dehydration of gel-forming nasal formulations, under biorelevant conditions, that could reduce mucosal tolerability of developed formulations.

The aim of this study was to develop a novel integrated approach enabling overall prediction of mucosal tolerability of swellable donepezil-loaded chitosan/mannitol microspheres previously developed by our group, throughout their interaction with the nasal mucosa, from initial contact to the expected residence period. These microspheres were originally prepared by ultrasonic spray-drying, a challenging and rarely used approach successfully employed to achieve the target particle size for nasal delivery [28]. In the present study, process reproducibility was assessed across independent batches alongside evaluation of mucosal tolerability. Attention was given to the role of mannitol as a key excipient in the microsphere formulation. Mannitol is widely used as a pharmaceutical excipient due to its favorable physicochemical properties such as low hygroscopicity, chemical inertness towards the active pharmaceutical ingredient, and excellent biocompatibility [29]. Owing to its osmotic activity, mannitol is also used as an active inhalation agent in patients with cystic fibrosis, where it increases mucus hydration, thereby enhancing mucociliary and cough clearance [30].

Current in vitro characterization of nasal powder formulations primarily focuses on aerosol performance, particle deposition, drug release, permeability, and mucoadhesion [31]. Beyond these conventional performance attributes, the hydration state of swellable nasal powder formulations is also an important consideration. The hygroscopic properties of individual excipients in powders intended for nasal administration have been investigated using dynamic vapor sorption [23], while swelling behavior of nasal powder formulations in contact with simulated nasal fluid has been evaluated using Franz diffusion cells [13,32]. In other pharmaceutical applications, gravimetric assays of evaporative water loss and water vapor transmission rate (WVTR) measurements have been employed to characterize water transport and dehydration behavior of hydrated formulations, particularly in the context of (trans)dermal delivery [33,34]. However, these approaches do not capture the complex water-transfer processes that may occur when a swellable nasal powder formulation is deposited onto the nasal mucosal surface. To address this gap, we developed a biomimetic experimental approach that enables integrated investigation of water evaporation, water transport through the swollen gel, resistance to dehydration, and rehydration through an available aqueous source under simulated nasal conditions. A climate chamber coupled with an immersion cell, serving as a formulation carrier, was used to establish a biomimetic model incorporating physiologically relevant temperature and humidity together with a mucin-coated interface. This setup enabled controlled simulation of water transfer between the mucosa and the formulation, allowing formulation-dependent differences in water exchange behavior to be characterized. Such differences may affect the hydration state and rheological properties of the swollen formulation, its water retention and behavior during nasal residence, as well as local mucosal hydration and, consequently, formulation performance and tolerability. Characterization of these processes is therefore relevant to the rational development and optimization of swellable nasal powder systems.

In this study, the proposed methodology was applied to donepezil-loaded chitosan microspheres, donepezil-loaded chitosan/mannitol microspheres and a corresponding donepezil-loaded chitosan microspheres/mannitol physical mixture. The biomimetic assessment of formulation behavior was complemented by solid-state and rheological characterization to elucidate the impact of formulation composition and the production process on the observed behavior. In addition, local tolerability was evaluated using the established slug mucosal irritation (SMI) assay. Together, these complementary biomimetic models provide mechanistic insight into formulation performance and enable prediction of local mucosal tolerability.

## 2. Materials and Methods

### 2.1. Materials

Donepezil hydrochloride (hereafter referred to as donepezil) was obtained from Carbosynth Ltd. (Compton, UK). Low-molecular-weight chitosan (50–190 kDa, 75–85% deacetylated; hereafter referred to as chitosan) was sourced from Sigma-Aldrich (Darmstadt, Germany). Mannitol was obtained from VWR International Ltd. (Lutterworth, Leicestershire, UK). Lactose monohydrate (GranuLac^®^; further denoted as lactose) was purchased from Meggle (Wasserburg am Inn, Germany). Simulated nasal fluid (SNF) was prepared by dissolving NaCl (Kemig, Zagreb, Croatia), KCl (Kemig, Zagreb, Croatia), and CaCl_2_·2H_2_O (Sigma-Aldrich, Darmstadt, Germany) in distilled water to a concentration of 150.0 mM, 40.0 mM and 5.3 mM, respectively. Mucin type III (bound sialic acid 0.5–1.5%, partially purified powder) was obtained from Sigma-Aldrich (Darmstadt, Germany).

All other chemicals and solvents used were of analytical grade and were purchased from Kemika (Zagreb, Croatia).

### 2.2. Methods

#### 2.2.1. Preparation of Microspheres

Microspheres were prepared according to the method published in our previous study [13]. Low-molecular-weight chitosan was selected based on preliminary formulation studies, as it provided optimal feed solution viscosity for ultrasonic spray-drying and produced microspheres with favorable particle size and flowability, which are critical quality attributes for nasal powder formulations. Briefly, donepezil-loaded microspheres containing mannitol or lactose as filler (D/C/M; D/C/L) were produced by spray-drying the aqueous solution containing donepezil, chitosan, and mannitol or lactose. First, a 1.5% (*w*/*w*) chitosan solution was prepared by dissolving chitosan in 0.5% (*V*/*V*) acetic acid at room temperature under continuous stirring for 24 h. Appropriate amounts of donepezil and mannitol or lactose were dissolved in previously prepared chitosan solution to obtain final concentrations of donepezil, chitosan and mannitol or lactose as follows: 0.4% (*w*/*w*), 1.5% (*w*/*w*) and 6.0% (*w*/*w*).

Microspheres were prepared using a Büchi Mini Spray Dryer B-290 (Büchi, Flawil, Switzerland) equipped with an ultrasonic nozzle (Büchi, Flawil, Switzerland). The following process parameters were used: aspirator rate of 100%, compressed air pressure of 5 bar, ultrasonic nozzle power set at 65%, inlet air temperature of 110 °C, outlet air temperature of 65 °C and feed pump rate of 10%. The spray-drying process was performed in open-loop mode using air as the drying gas. The process yield was calculated as the ratio of the mass of collected spray-dried microspheres to the total initial mass of solids in the feed solution. As the recovered material was not dried prior to weighing, the reported process yields include residual moisture, which may result in slightly higher values than dry matter yields.

Corresponding donepezil-loaded chitosan microspheres (D/C) were prepared under the same donepezil and chitosan feed concentration and identical spray-drying conditions as used for D/C/M and D/C/L, differing only in the inlet temperature, which was set to 140 °C.

#### 2.2.2. Preparation of the Powder Blend

Powder blend was prepared by mixing the D/C microspheres with mannitol as a carrier with particle size between 45 and 63 μm. This particle size of the carrier was obtained using a laboratory sieve shaker (Vibratory Sieve Shaker AS 200, Retsch^®^, Haan, Germany) equipped with 20 cm sieves (Retsch^®^, Haan, Germany; nominal apertures 63 and 45 μm). Mannitol was sieved for 10 min at 50% amplitude. Powder blend was then prepared by mixing D/C microspheres with mannitol at the same weight ratio as in the D/C/M sample, obtaining a D/C + M sample. Following a 5 min premixing step on an MX-S vortex mixer (1250 rpm; DLAB Scientific Co., Ltd., Beijing, China) in a 50 mL centrifuge tube (Falcon^®^, Corning Costar Inc., Corning, NY, USA), the mixture was further blended using a Turbula^®^ mixer (WAB Group, Muttenz, Switzerland) for 10 min at 70 rpm. The composition of spray-dried microspheres (D/C, D/C/M and D/C/L) and powder blend D/C + M is given in Table 1.

Blend homogeneity was assessed by determining drug content in 15 mg powder blend samples collected from the top, middle, and bottom of each centrifuge tube. The samples were dissolved in 25.0 mL of distilled water, ultrasonicated for 2 h to allow complete drug release and dissolution, stirred on a magnetic stirrer for 30 min and afterwards filtered (0.2 μm pore size) and analyzed for donepezil content by high-performance liquid chromatography (HPLC) as described in Section 2.2.4. All measurements were performed in triplicate.

Adequate homogeneity was defined as a mean percentage ratio of experimentally determined to theoretical donepezil mass within 100.0 ± 5.0%, with a relative standard deviation (RSD) ≤ 5.0% [35].

#### 2.2.3. Determination of Entrapment Efficacy and Donepezil Content in Microspheres

Entrapment efficacy and donepezil content in microspheres were determined as described previously [13]. An accurately weighed amount of microspheres (10 mg) was quantitatively transferred into a 25.0 mL volumetric flask and diluted to volume with purified water. The dispersion was placed in an ultrasonic bath for 2 h and subsequently stirred on a magnetic stirrer for another 30 min. Prior to analysis, the dispersions were filtered through a membrane filter with a pore size of 0.20 μm.

Entrapment efficiency and donepezil content in the spray-dried microspheres were determined using the HPLC method described in Section 2.2.4. The donepezil concentration in each sample was calculated from the corresponding calibration curve. Based on these values, the entrapment efficiency (EE, %) was calculated using the following Equation (1):

(1) $$EE\left(\%\right)=\frac{actual\;drug\;content\;(mg)}{theoretical\;drug\;content\;(mg)}\times 100$$

The drug loading (DL, %), expressed as the mass fraction of donepezil in the microspheres (*w*/*w*), was calculated using Equation (2) as follows:

(2) $$DL\left(\%\right)=\frac{mass\;of\;encapsulated\;drug\;(mg)}{mass\;of\;microspheres\;analysed\;(mg)}\times 100$$

#### 2.2.4. Quantitative Analysis of Donepezil

Quantitative analysis of donepezil was performed using an HPLC method on a 1260 Infinity II LC system (Agilent Technologies, Santa Clara, CA, USA), as described previously [13]. Chromatographic data were processed using OpenLab software (Agilent Technologies, Santa Clara, CA, USA). Separation was achieved on a Kinetex C18 reverse-phase column (250 × 4.6 mm, 2.6 μm particle size) equipped with a corresponding guard column, both supplied by Phenomenex (Torrance, CA, USA).

The method was based on Pappa et al. [36], with minor modifications. In brief, the mobile phase consisted of 0.02 M phosphate buffer (pH 2.7), methanol, and triethylamine mixed in a 50:50:0.5 (*V*/*V*/*V*) ratio. Further on, the flow rate was 1.0 mL min^−1^ and the injection volume was 20 μL. Analysis was performed at 25 °C, with detection wavelength at 268 nm. The total run time was 7 min, and donepezil eluted at approximately 5 min. The method was validated according to the International Conference on Harmonization (ICH) guideline Q2 (R1) [37].

#### 2.2.5. Particle Size Distribution

The laser diffraction method (previously developed in [13]) was used to determine the particle size distribution of the prepared microspheres. Malvern Mastersizer 3000 (Malvern Instruments Ltd., Worcestershire, UK) equipped with a 300 mm focal-length lens was used. The instrument featured a Hydro SV dispersion unit with a magnetic stirrer. Approximately 5 mg of microspheres were dispersed in ~10 mL of 96% (*V*/*V*) ethanol and ultrasonicated for 10 min to obtain a homogeneous suspension. A background measurement was recorded prior to sample analysis. The microsphere suspension was then introduced into the Hydro SV cell until an obscuration level of 10–20% was reached. Samples were equilibrated for 30 s before measurement, and each was analyzed in pentaplicate. Data analysis was based on Mie scattering theory using an refractive index of 1.460, 1.630 and 1.560 for samples D/C/M, D/C/L and D/C, respectively. The results are reported as volume-based diameters *D*_v_10, *D*_v_50, and *D*_v_90. The volume percentage of particles smaller that 10 µm is also reported.

#### 2.2.6. Solid-State Analysis

Differential scanning calorimetry (DSC) was performed using a Perkin-Elmer Diamond DSC instrument (PerkinElmer Inc., Waltham, MA, USA), calibrated with indium (99.98% purity; melting point: 156.61 °C; fusion enthalpy: 28.71 J g^−1^). Samples (2–5 mg) were accurately weighed using a Mettler M3 microbalance (Mettler-Toledo, Gießen, Germany) and sealed in aluminum pans with pierced lids. Measurements were carried out under a nitrogen purge (25 mL min^−1^) at a heating rate of 10 °C min^−1^ over the temperature range of 25–300 °C.

X-ray powder diffraction (XRPD) patterns of the initial compounds and prepared samples were recorded using a Malvern Panalytical Aeris X-ray diffractometer (Malvern Panalytical Ltd., Malvern, UK). Samples were evenly spread on a silicon zero-background holder to minimize background interference. Data were collected over a 2θ range of 5–40° using a continuous scan mode at a rate of 7° min^−1^. The instrument was equipped with an X-ray tube with a copper anode (Cu Kα radiation: λ(Kα_1_) = 1.54056 Å; λ(Kα_2_) = 1.54439 Å; Kα_1_/Kα_2_ intensity ratio = 0.5). The tube operated at 40 kV and 15 mA. Measurements were performed at ambient temperature under atmospheric conditions.

#### 2.2.7. Assessment of Water Loss from and Through the Gel by Evaporation

**Figure 2:**
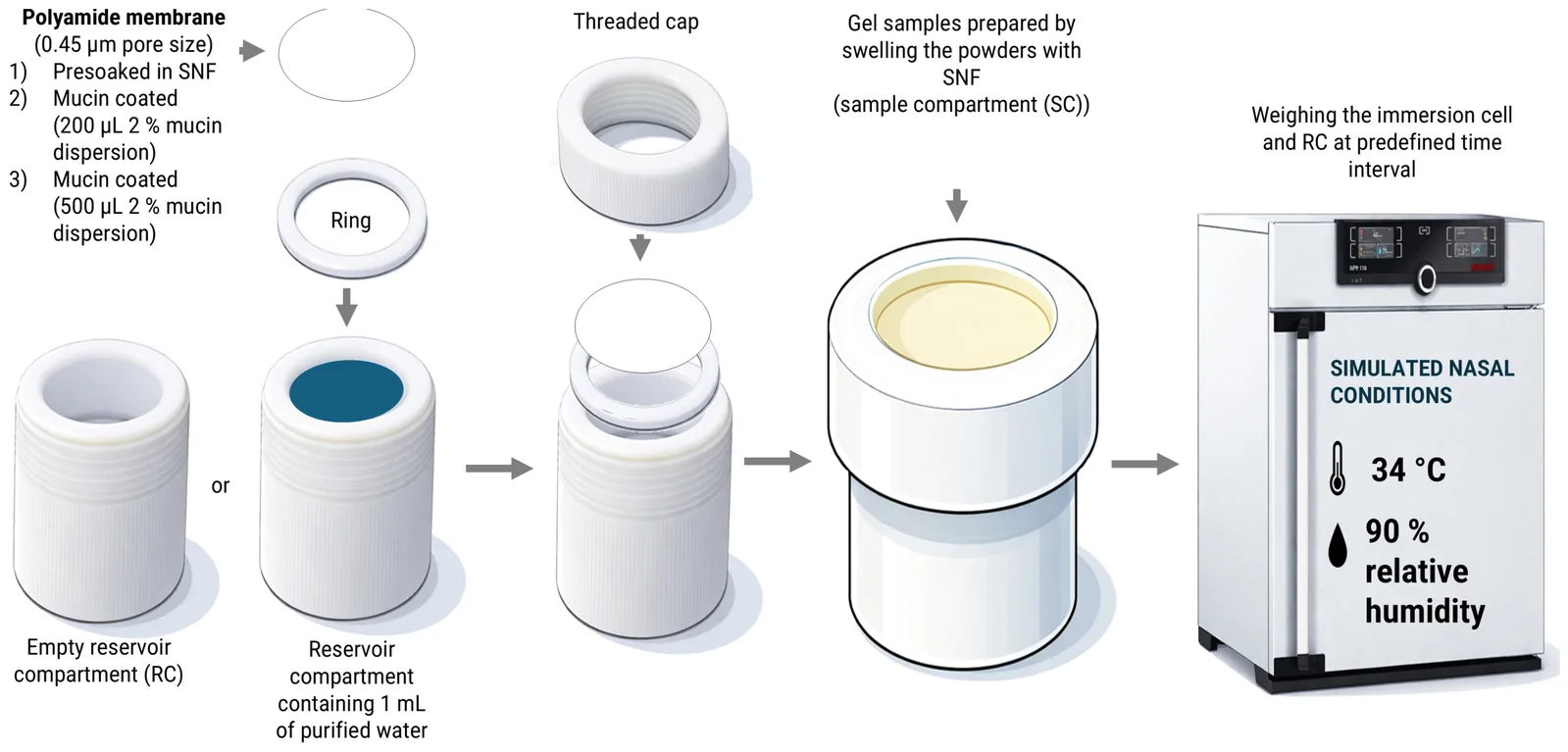
Assessment of water loss from and through the gel by evaporation. Figure is created with Illustrae.co (UK) and Inkscape.com (USA).

The assessment of water loss through and from the gel (formed by powder swelling in SNF) was performed using polytetrafluoroethylene dissolution Enhancer (immersion) cells with a surface area of 4 cm^2^ and adjustable volume (Agilent Technologies, Santa Clara, CA, USA). Originally, Enhancer cells were designed for evaluating drug release from topical formulations [38]. The cells consist of a cylindrical reservoir compartment (RC), a ring, and a threaded cap with a circular opening (Figure 2). The volume of RC was set at 3.0 mL.

In the experimental setup, the RC was prepared either empty or containing 1 mL of purified water. The ring was placed onto the reservoir, followed by positioning a polyamide membrane (pore size 0.45 µm; Sartorius Stedim Biotech GmbH, Göttingen, Germany) cut to the dimensions of the ring. Two types of membranes were used: uncoated and mucin-coated. Mucin coating was achieved by applying 200 or 500 μL of 2% (*w*/*w*) mucin aqueous dispersion and drying for 12 h. Prior to experiments, all membranes (uncoated and coated) were presoaked in SNF. The system was then sealed with the lid containing the circular opening.

Gel samples prepared by swelling the powders with SNF were applied onto the exposed surface of the polyamide membrane (sample compartment) and smoothed to the level of the lid using a metal spatula. Powder systems D/C, D/C/M and D/C + M were used for gel preparation. The powder systems were mixed with SNF in a laboratory beaker using a metal spatula until a homogeneous gel was obtained. The volume of SNF used for gel preparation was 1 mL. More specifically, the amount of microspheres used to prepare D/C/M and D/C gel corresponded to the quantity that absorbs 1 mL of SNF during the swelling process (100 and 29 mg, respectively; Table 2). The amounts of D/C and M in D/C + M powder mixture used to prepare D/C + M gel were defined to match that of D/C/M (powder mixture of 24 mg D/C and 76 mg of M; 100 mg in total; Table 2). The swelling of chitosan and chitosan–mannitol microspheres loaded with donepezil was described previously [13].

The fully assembled cell, comprising the gel sample, threaded cap, membrane (uncoated or coated), and empty RC, was weighed on an analytical balance prior to the placement in the climate chamber. The climate chamber was set to simulate nasal conditions (34 °C and 90% relative humidity [39,40]). After introduction into the chamber, the cell was weighed at 1.5 h intervals over a total period of 7.5 h. Water loss from the gel was determined based on the recorded changes in mass.

For experiments conducted with water present in the RC, the fully assembled cell and the RC containing water were weighed using an analytical balance prior to the experiment and during thermostating at 1.5 h intervals in the climate chamber. Changes in mass were used to monitor variations in water content in both the RC and the gel sample positioned above the water over time. Water loss through and from the gel was monitored using the experimental setup shown in Figure 2 and described in Table 3. All experiments were performed in triplicate, and appropriate controls were included for both experimental configurations (empty and RC containing water), as detailed in Table 3.

#### 2.2.8. Rheological Characterization of Gels

Gels for rheological characterization were prepared by swelling D/C/M, D/C + M and D/C samples with appropriate amount of SNF in a laboratory beaker, as described previously in Section 2.2.7. Additionally, two more concentrated D/C/M gels were prepared by swelling the D/C/M microspheres in a reduced amount of SNF reflecting gel dehydration level observed after 2 h and 7.5 h in water loss studies conducted using empty RC and an uncoated membrane.

The rheological properties of the prepared swollen powder samples were evaluated using a Modular Compact Rheometer (MCR 102; Anton Paar GmbH, Graz, Austria) equipped with a Peltier temperature control system. Oscillatory rheological measurements were conducted using a parallel-plate geometry (PP25, 25 mm diameter), while rotational measurements were performed using a cone-plate geometry (CP25, 25 mm diameter, 1° cone angle). Rheological data were analyzed using RheoCompass™ Light software (Version 1.23.403; Anton Paar GmbH, Graz, Austria).

##### Rotational Test

Flow behavior of the prepared samples was evaluated by rotational flow curve test using a CP25 at 34 °C. The zero gap between the measuring body and the lower plate was 0.049 mm. Apparent viscosity was measured as a function of shear rate over the range of 0.1–1000 s^−1^. All measurements were performed in duplicate.

##### Oscillatory Tests

Oscillatory rheological measurements were performed using a PP25, at 34 °C, with the measuring gap fixed at 0.500 mm. Each formulation was analyzed in duplicate.

An amplitude sweep test was first conducted to determine the linear viscoelastic region (LVR) of the gels. The storage modulus (*G*′) and loss modulus (*G*″) were recorded as a function of shear strain over the range of 0.1–1000% at a constant angular frequency of 6.28 rad·s^−1^.

Subsequently, frequency sweep tests were performed within the LVR (strain applied at 1%) to further characterize the viscoelastic behavior of the formulations. *G*′ and *G*″ were recorded over the range of angular frequencies of 1 to 100 rad·s^−1^.

#### 2.2.9. Slug Mucosal Irritation Assay

The slug mucosal irritation (SMI) assay was employed to assess the potential irritancy of the powders on nasal mucosa. The procedure followed the method described by Trenkel and Scherließ [23]. Slugs of the species *Arion lusitanicus* were collected by wild harvesting. Two days before the experiment, slugs weighing 3–6 g were individually isolated on paper towels moistened with phosphate-buffered saline (PBS, pH 7.4). During that period, the body wall of each slug was wetted daily with 1 mL of PBS and inspected for signs of mucosal damage. At the start of the experiment, each slug’s body weight (BW) was recorded.

A total of 50 mg of the tested powder formulation was placed in a Petri dish, and the combined mass of the dish and sample was noted. Slugs were then positioned on the powder formulation for a 15 min contact period (CP). After each CP, slugs were transferred to another dish containing 1.5 mL of phosphate-buffered saline (PBS) for a 60 min resting period. During this time, the initial dish containing the formulation and mucus produced during the CP was weighed.

This sequence was repeated twice more, resulting in three contact periods in total. Total mucus production (TM) after the three CPs was calculated using Equation (3):

(3) $$TM\left(\%\right)=\frac{\Sigma M(mucus\;per\;CP,g)}{BW\;(g)}\times 100\%$$

The same procedure was performed for the negative control (100 µL PBS) and the positive control (100 µL of 1% *w*/*V* benzalkonium chloride, BAC). All experiments were conducted in triplicate, using a different slug for each replicate.

#### 2.2.10. Statistical Analysis

Statistical analysis of the results on the slug mucosal irritation assay and dehydration studies was performed using GraphPad Prism 8.0.2. (trial version), employing a one-way analysis of variance (ANOVA) followed by Tukey’s post hoc test, with *p* < 0.05 set as the minimal level of significance.

## 3. Results and Discussion

Building on our previous work demonstrating the potential of chitosan/mannitol microspheres for safe and effective nose-to-brain delivery of donepezil, the aim of this study was to develop a novel experimental approach to evaluate and predict nasal mucosal tolerability of this swellable nasal powder platform. A biomimetic method was developed to monitor water content changes in swollen microspheres under simulated nasal conditions to predict the dehydration potential of donepezil-loaded nasal powders.

### 3.1. Preparation of the Powder Samples

Donepezil-loaded chitosan and chitosan/mannitol microspheres were prepared by spray-drying using an ultrasonic nozzle. Microspheres were prepared using low-molecular-weight chitosan providing optimal atomization, particle size and powder flowability [13].

To evaluate mannitol as a critical excipient, a lactose-based formulation was included in this study as a comparative control. Process reproducibility was evaluated by repeating the spray-drying procedure three times, resulting in three independent batches [41,42]. Batch-to-batch variability was assessed by calculating relative standard deviation (RSD, %: Table 4).

Particle sizes for the D/C and D/C/M samples measured in the present study were in line with the particle size distribution reported in our previous study [13]. While D/C microspheres did not meet the regulatory size requirements for nasal powders, the D/C/M microsphere size distribution complied, with only 7.0 ± 1.4% sample volume comprising particles smaller than 10 μm. Moreover, in our previous study, the D/C/M formulation demonstrated appropriate deposition performance in an anatomically relevant in vitro nasal cast model, with the entire delivered dose being deposited within the nasal cavity and 65.5% reaching the olfactory region [13]. These findings indicate that the formulation exhibited suitable aerodynamic behavior for efficient localized nasal drug delivery. D/C/L microspheres exhibited significantly smaller particle sizes than the corresponding D/C/M sample (prepared using the same drug/chitosan/filler ratio and spray-drying conditions), with 37.0 ± 8.6% sample volume comprising particles smaller than 10 μm (Table 4).

A similar trend was reported by Almansour et al. [43], who observed smaller spray-dried lactose-based microparticles in relation to mannitol-based formulations. One possible explanation lies in the different solidification behavior of the two excipients during spray-drying. Mannitol readily crystallizes during droplet drying, promoting early shell formation and the development of relatively large, porous particles [44]. In contrast, lactose has a much higher glass-forming tendency and is predominantly recovered in an amorphous state, with crystallization strongly dependent on the drying conditions [45].

Batch-to-batch variability was generally low, with comparable RSD values for D_v_50 (13.7–15.9%), the fraction of particles below 10 µm (20.2–24.0%), and consistently low variability in drug loading and entrapment efficiency across all formulations. Greater variability was observed for D_v_10 (9.6% to 19.1%) and particularly D_v_90 (8.4% to 65.6%). RSD analysis showed that D/C/M microspheres exhibited generally comparable reproducibility to D/C/L microspheres. However, the volume fraction of particles smaller than 10 μm was significantly lower for D/C/M than for D/C/L microspheres (Table 4).

This work further confirms the suitability of an ultrasonic nozzle for the one-step production of nasal microspheres with controlled particle size and built-in quality attributes. The results demonstrate that, although appropriate process parameters are essential, formulation composition, particularly the use of mannitol as a critical excipient, plays a dominant role in achieving controlled particle size at the investigated chitosan/filler/donepezil weight ratio.

To evaluate whether the presence of mannitol and formulation processing influence the potential sensory effects of donepezil-loaded nasal powders at nasal mucosa, D/C, D/C/M and D/C + M were compared in the subsequent studies. The powder blend sample showed adequate homogeneity of 99.6 ± 4.4%.

### 3.2. Results of Solid-State Analysis

Differential scanning calorimetry (DSC) is a widely used, rapid thermal analysis method well suited for early-stage solid-state characterization, especially valuable in assessing solid dispersions, microspheres, and other solid-dosage forms. This indirect technique measures heat flow to determine solid-state properties such as crystallinity, polymorphism, and miscibility. Nonetheless, the accuracy and interpretation of DSC data can be significantly influenced by factors such as heating and cooling rates, sample preparation, moisture levels, and the sample’s thermal history [46]. To ensure reliable insights into the solid-state characteristics of the tested materials, it is recommended to complement DSC with other nonthermal methods, such as X-ray powder diffraction (XRPD).

**Figure 3:**
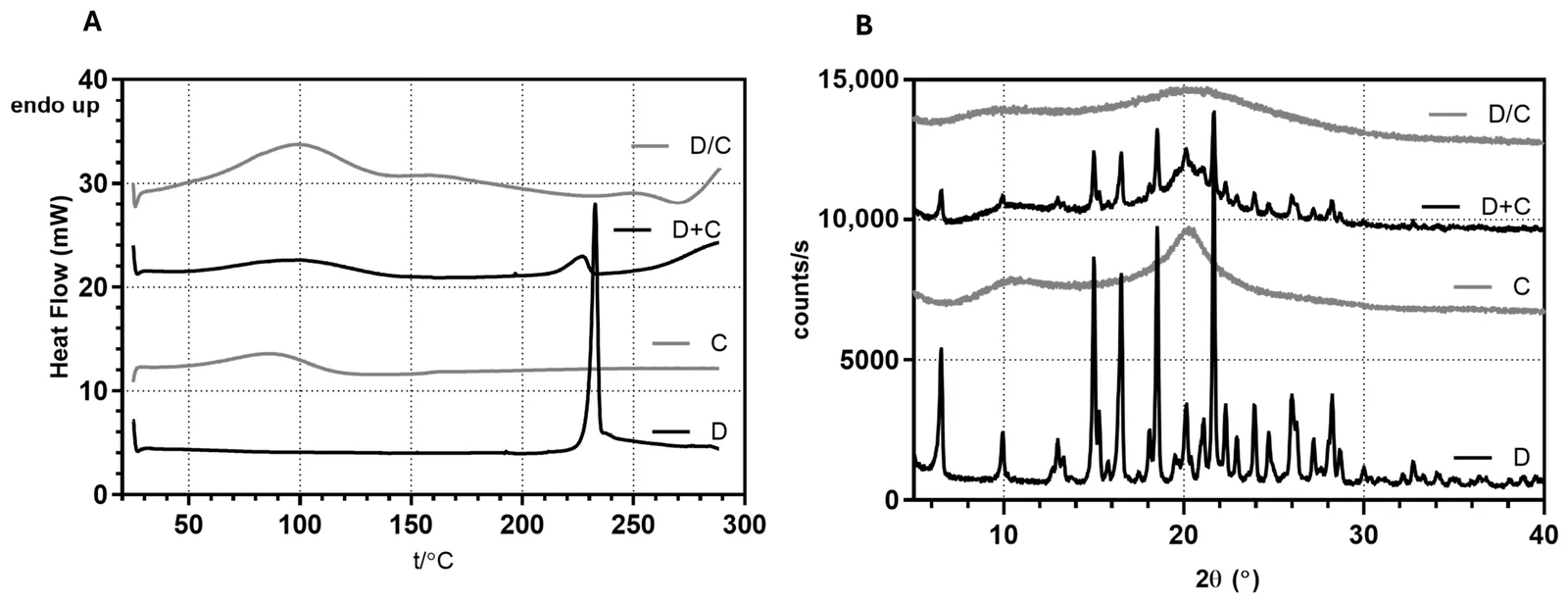
DSC thermograms (**A**) and XRP diffraction pattern (**B**) of donepezil (D), chitosan (C), their physical mixture (D + C) and spray-dried microspheres (D/C).

DSC analysis of donepezil hydrochloride (Figure 3) exhibited a sharp endothermic peak with the onset temperature of 229.78 °C, corresponding to the melting of the thermodynamically most stable polymorph III (Δ*H*_fus_ = 129.18 J/g) [47]. The XRPD diffractogram showed the most prominent peaks at 6.56°, 10.00°, 18.50°, and 21.66° 2*Θ*, which are typical of polymorphic form III of the drug [48]. The DSC thermogram of chitosan showed a broad endothermic event in the 50–120 °C range, attributed to moisture loss, while XRPD exhibited a halo effect, characteristic of an amorphous polymer. In the DSC thermogram of the physical mixture of donepezil hydrochloride and chitosan, the typical endothermic events were still present but shifted to lower temperatures (*Tonset* = 217.24 °C) and exhibited reduced intensity, consistent with the drug content in the sample. XRPD corroborates this observation by showing the superposition of the respective diffraction patterns of each compound. In the DSC thermograms of spray-dried microspheres, the characteristic drug melting peak is absent, indicating that the drug has been amorphized within the polymeric matrix during the spray-drying process. Furthermore, XRPD analysis also confirmed the amorphous nature of the encapsulated drug.

**Figure 4:**
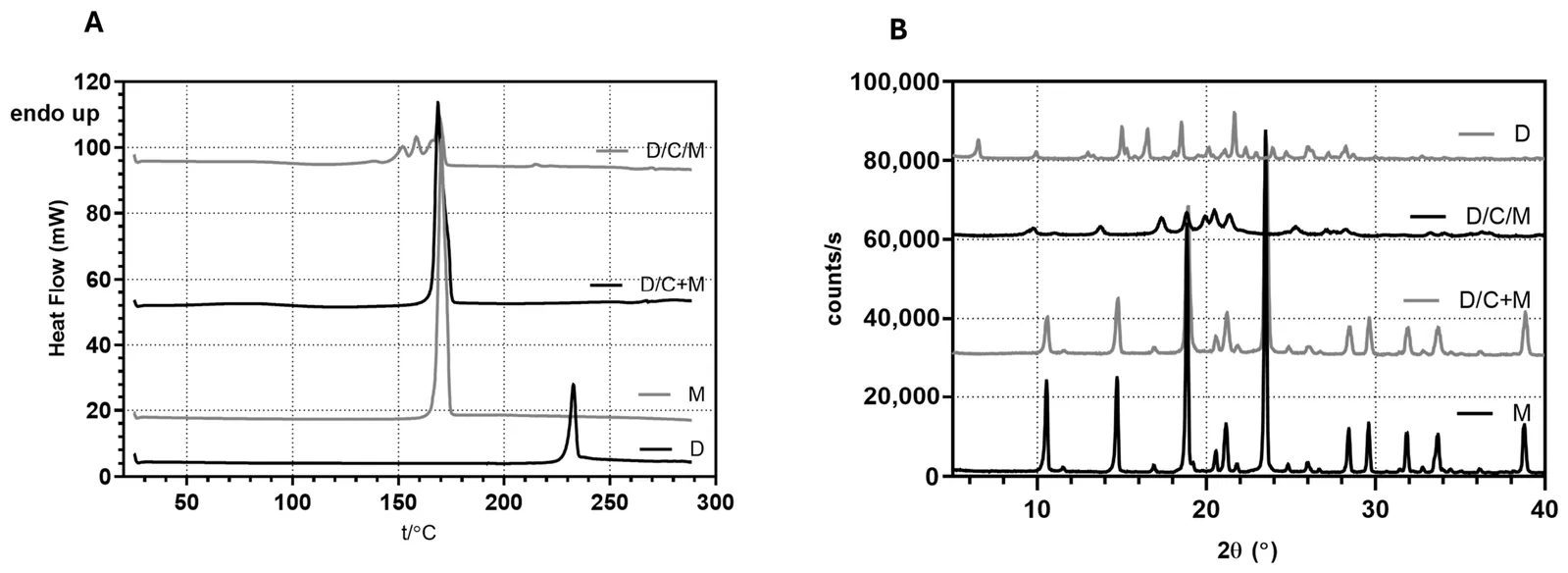
DSC thermograms (**A**) and XRPD pattern (**B**) of samples with mannitol: donepezil (D), mannitol (M), spray-dried chitosan microsphere physical mixture with mannitol (D/C + M), and spray-dried composite microspheres (D/C/M).

The results of the solid-state characterization of the mannitol-containing samples are presented in Figure 4. In the DSC thermogram, mannitol exhibited a sharp melting peak with an onset at 166.51 °C, consistent with the β-polymorphic form of mannitol. This was further confirmed by XRPD analysis, which showed characteristic diffraction peaks at 10.4°, 14.6°, and 23.4° 2*Θ*, in agreement with previously reported values for β-mannitol [49]. In the physical mixture of chitosan microspheres and mannitol, the typical melting behavior and XRPD peaks of β-mannitol remained clearly detectable, indicating the absence of any solid-state interactions or polymorphic transformations caused by mixing. In contrast, the DSC thermogram of composite spray-dried microspheres showed several endothermic peaks in the temperature range of 146.76 to 174.58 °C. In DSC scans, the metastable δ-form of mannitol often transforms into the α- or β-form during heating, resulting in multiple or shifting peaks [49]. Milenkova et al. demonstrated that such phase transitions also occur in the spray-dried microparticles containing chitosan [50]. Further insight into the mannitol solid phases present in the composite microspheres was obtained by XRPD, where peaks at 2Θ value of 9.35°, 18.82° and 20.43° are typical for metastable δ-form, while peaks at 13.43° and 17.05° indicate the simultaneous presence of α-mannitol form [49]. Peaks typical of β-form, especially a high-intensity peak at 23.4° 2*Θ*, were not observed. The simultaneous presence of δ- and α-mannitol is often observed in co-spray-dried products, where the content of each mannitol form depends on the processing parameters applied [51]. In addition, the simultaneous presence of δ- and α-mannitol may have implications for the long-term physical stability of the spray-dried product. Recent studies have demonstrated that the presence of an active pharmaceutical ingredient may promote the formation and persistence of δ-mannitol in co-spray-dried systems [51]. Accordingly, it may be hypothesized that donepezil plays a similar stabilizing role in the developed microparticles, facilitating the persistence of δ-mannitol within the solid matrix. In addition, certain polymers, such as polyvinylpyrrolidone (PVP), can promote the formation and stabilization of metastable mannitol polymorphs [52]. Therefore, the presence of chitosan in the present formulation may further contribute to the kinetic stabilization of δ-mannitol by restricting molecular mobility and inhibiting crystal phase transformation. We have analyzed the stability of the D/C/M sample (stored in airtight containers in the refrigerator at 4 °C). As presented in the Supplementary Files (Figure S1), the results confirmed the long-term stability of the tested D/C/M sample and verified the above-mentioned hypothesis: coexistence of α- and δ-mannitol in the developed microparticles reflect a kinetically stabilized polymorphic system resulting from the combined effects of formulation composition and spray-drying process conditions.

### 3.3. Assessment of Gel Dehydration via Evaporative Water Loss Under Simulated Nasal Conditions

Water loss from the gel and through the gel was examined for donepezil-loaded chitosan and chitosan/mannitol microspheres (D/C and D/C/M, respectively), as well as for the corresponding control physical mixture of D/C microspheres with mannitol (D/C + M) (Table 3). Measurements were performed over 7.5 h under controlled environmental conditions (34 °C, 90% RH) designed to mimic the physiological environment of the human nasal cavity [39,53].

For gel preparation, powder samples were allowed to swell in 1 mL of SNF. The masses of the D/C and D/C/M microspheres were predetermined based on previous swelling studies [13], corresponding to the amount required to absorb 1 mL of SNF. The mass of the control physical mixture (D/C + M) was adjusted to match that of D/C/M microspheres, ensuring consistent SNF content and comparable amounts of donepezil and chitosan across all gels. The compositions of gels are summarized in Table 2.

Gel dehydration via evaporative water loss under simulated nasal conditions was assessed using two setups: (i) gels positioned above a dry reservoir compartment and (ii) gels above a water-containing reservoir compartment, with water losses from the gel and reservoir quantified separately. In all experiments, gels were placed on the SNF-presoaked polyamide membrane. To account for the potential influence of gel–mucin interactions on gel layer dehydration, experiments were conducted using a dry reservoir compartment and a mucin-coated polyamide membrane simulating contact with the nasal mucosa. Two mucin coating thicknesses enabled assessment of the model’s ability to discriminate between formulation- and barrier-dependent effects on the dehydration profile.

**Figure 5:**
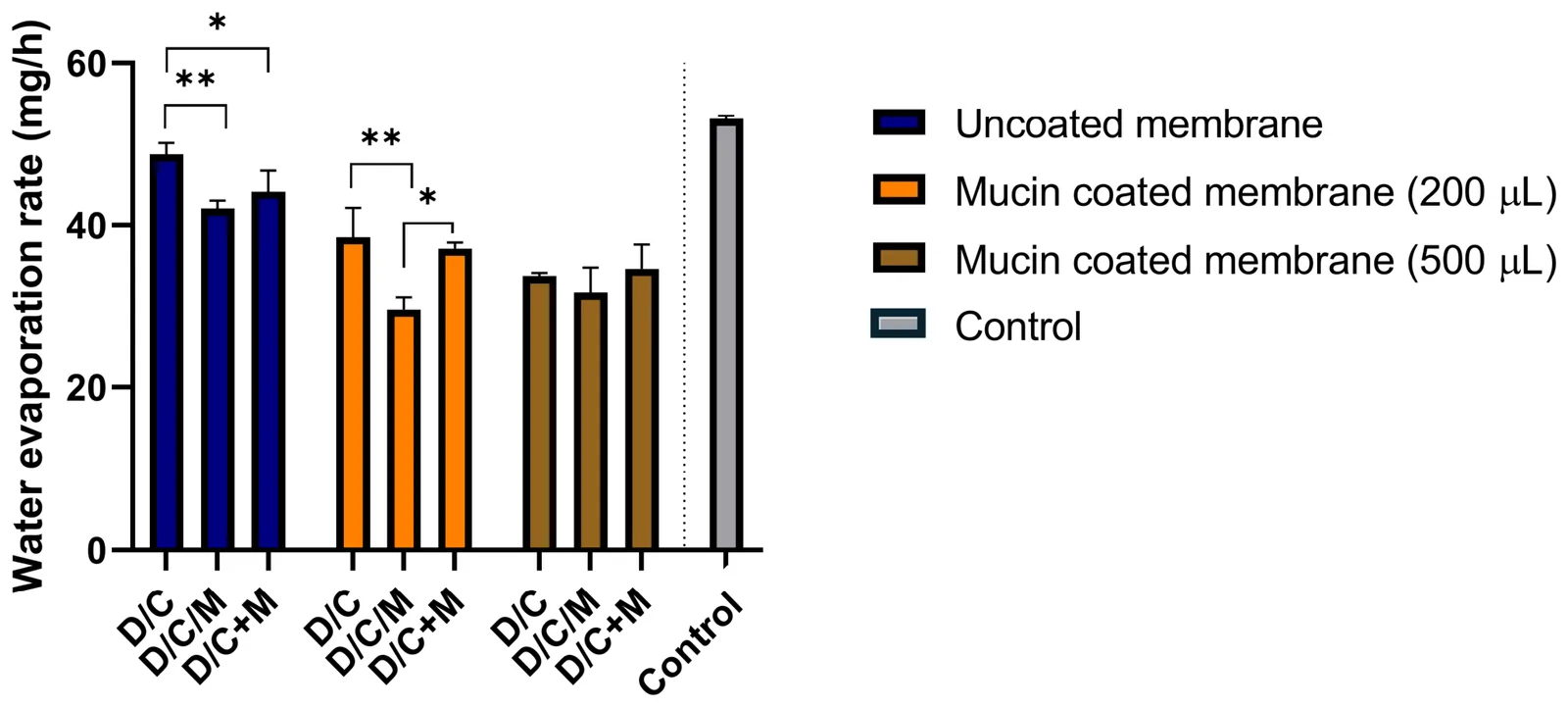
Water evaporative loss from the gel measured using uncoated and mucin-coated membranes and empty reservoir compartment. Values are mean ± SD (*n* = 3). *—statistically significant difference, *p* < 0.05; **—statistically significant difference, *p* < 0.01.

**Figure 6:**
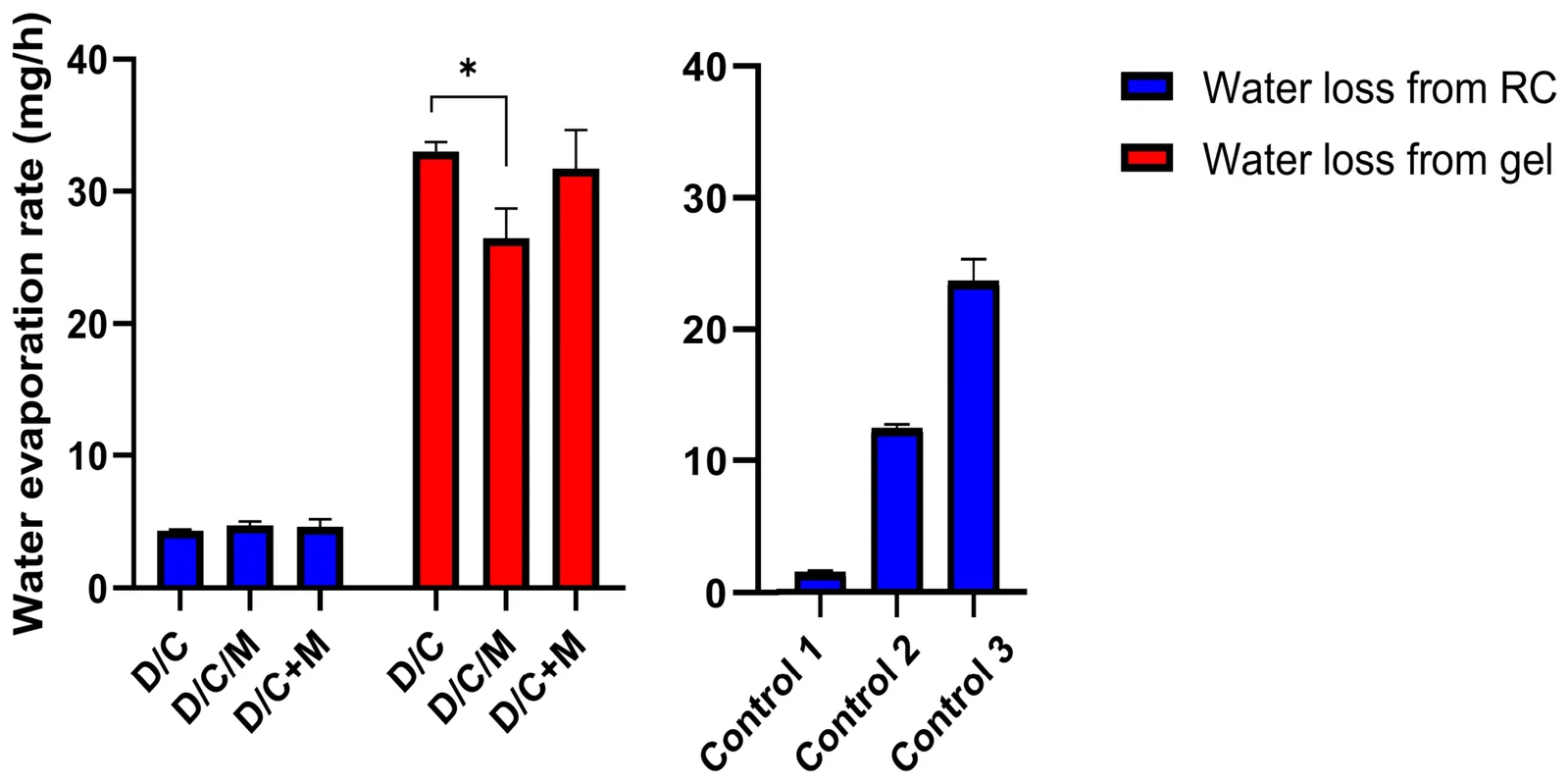
Water evaporative loss from the gel and from the reservoir compartment (RC) measured using uncoated membrane and RC containing 1 mL of water. Controls refer to water evaporation rate from RC through nonpermeable Vaseline-coated membrane (control 1); SNF-presoaked noncoated membrane (control 2) and without a membrane (control 3). Values are mean ± SD (*n* = 3). *—statistically significant difference, *p* < 0.05.

In all experiments, cumulative evaporative water loss (from the gel and reservoir compartment) increased linearly with time (R^2^ was predominantly above 0.99). The slopes obtained from the corresponding linear regressions, representing water evaporation rates (WER) under the test conditions, were calculated for each sample and experimental setup. The mean slope ± standard deviation across replicates is presented in Figure 5 and Figure 6.

For gels above a dry reservoir compartment, WER ranged from 29.6 ± 1.5 to 48.7 ± 1.4 mg h^−1^, corresponding to 5.9–9.7% water loss within 2 h, the anticipated residence time of a powder formulation on the nasal mucosa [54]. Based on visual inspection after 2 h of dehydration, the chitosan-based gels maintained hydrated appearance, without clear signs of drying or film formation, which is advantageous for minimizing nasal mucosal irritation and supporting patient adherence. Controlling gel dehydration is also important to prevent excessively high viscosity that could impair mucociliary function and disrupt respiratory homeostasis [55]. Water in hydrogels is commonly described as existing as free, intermediate, and bound fractions that differ in strength of interaction with the polymer matrix and mobility [56,57]. Water molecules interacting with polymer functional groups through hydrogen bonding are retained longer during dehydration than bulk-like water. In chitosan-based hydrogels, this behavior is particularly pronounced due to its unique molecular structure. Chitosan contains a high density of hydroxyl and primary amino groups capable of forming extensive hydrogen-bond networks with water molecules, while its cationic nature enables additional electrostatic interactions under physiological conditions [58,59,60].

Applying the mucin coating onto the membrane significantly reduced WER (Figure 5), confirming mucin’s water-retention capacity. Mucin is a highly glycosylated polymeric glycoprotein whose carbohydrate side chains strongly interact with water through hydrogen bonding, forming highly hydrated glycan domains and dynamic supramolecular networks capable of retaining substantial amounts of water [61,62,63]. The observed reduction in WER could be explained by the interaction of mucin with both chitosan and the free water present in the gel, reducing water mobility. As a result, the mucin can contribute to hydration reservoir effect and diffusion barrier properties, slowing water transport and evaporation.

For gels on an uncoated mucin membrane above a water-containing reservoir compartment, WER ranged from 26.4 ± 2.3 to 33.0 ± 0.7 mg h^−1^ (Figure 6), representing a decrease of approximately 29–37% compared with the corresponding WER values obtained using a dry reservoir compartment. This experimental setup demonstrated gel rehydration, simulating water transfer from the underlying mucosal surface. In parallel, WER from the reservoir compartment indicated a moderate occlusive effect for all formulations relative to the Vaseline-coated (positive control) and the SNF-presoaked (negative control) membranes, suggesting no risk for mucosal dehydration (Figure 6).

Across all experimental setups (except at higher mucin coating thickness), WER from the gel decreased in the order WER_D/C_ > WER_D/C+M_ > WER_D/C/M_. Chitosan-based gels undergo rapid and pronounced swelling upon contact with aqueous media due to low crosslinking density and large numbers of hydrophilic amino and hydroxyl groups, enabling substantial water uptake within the three-dimensional gel network [64]. The incorporation of mannitol markedly reduced the WER from the gels. As a polyol containing multiple hydroxyl groups, mannitol exhibits strong water-retention capacity, thereby contributing to the maintenance of gel hydration. In polymer hydrogels, confinement within the network and interactions with polymer chains lead to heterogeneous hydration domains whose hydrogen-bond structure and molecular dynamics differ from those of bulk water [65,66,67]. As a result, water molecules involved in stronger hydrogen-bond interactions and interfacial confinement require higher energy for removal, leading to increased enthalpy of desorption compared with bulk-like water. Mannitol can further modify this hydration structure. It can form multiple hydrogen bonds with water molecules and polymer chains and may increase the fraction of less mobile water within the hydrogel.

Polyols are also described as kosmotropic solutes capable of stabilizing hydrogen-bond networks in water [68]. This effect is supported by experimental and spectroscopic studies showing that polyols reorganize the hydrogen-bond network and slow water dynamics, leading to more structured hydration environments [69,70,71,72,73]. Complementary spectroscopic and theoretical analyses further indicate that polyols can form cooperative hydrogen-bond networks with surrounding water molecules, stabilizing local hydration structures [74]. From a thermodynamic perspective, such structured and dynamically constrained hydration domains are associated with stronger intermolecular interactions, meaning that additional energy is required to disrupt these hydrogen-bond networks. Consequently, water confined within these environments may exhibit higher enthalpy of desorption (or evaporation) compared with bulk-like water, which contributes to reduced water mobility and a slower rate of water loss from the hydrogel.

As indicated above, lower WER was observed for D/C/M gel in comparison to D/C + M gel, despite the same constituent concentrations. The difference in WER may be attributed to different preparation methods of corresponding precursor powder systems. Namely, it is likely that spray-drying-related pre-existing molecular interactions enhanced water retention within the D/C/M gel matrix, thereby minimizing the risk of surface crust formation during dehydration.

At higher mucin coating thickness, the effect of mannitol presence and/or formulation processing history on the WER from the gels was no longer observed likely due to the dominant water-binding capacity of mucin. In contrast, thinner mucin coating enabled the in vitro biobarrier to discriminate between formulation- and barrier-dependent effects, showing the potential for use in nasal formulation screening studies.

### 3.4. Rheological Properties of Gels: Effects of Mannitol and Dehydration

**Figure 7:**
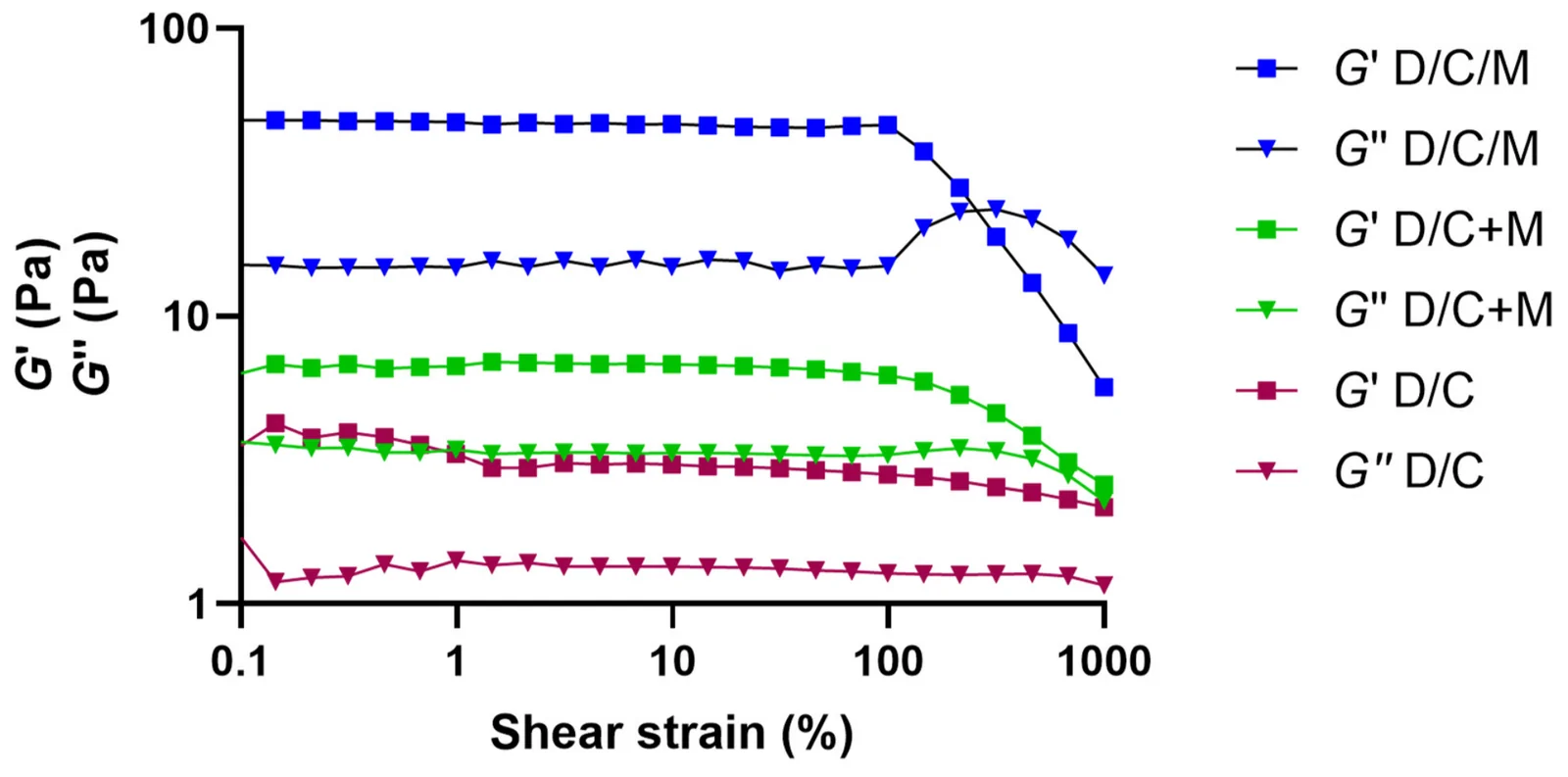
Amplitude sweep test performed on gels prepared by swelling the samples D/C/M, D/C + M and D/C in SNF.

The first goal of rheological studies was to evaluate the effect of mannitol presence on the physical properties of the gel formed by the powder systems. The gels were prepared by swelling the D/C microspheres, D/C/M microspheres and D/C + M physical mixture in a predetermined amount of SNF [13], as explained in Section 2.2.7. The storage modulus (*G*′) as a measure of resistance to elastic deformation, and loss modulus (*G*″) as a measure of resistance to viscous flow, were measured in oscillatory amplitude sweep test as functions of shear strain. The results are presented in Figure 7.

All samples exhibited a stable gel structure, as *G*′ exceeded *G*″ values. The storage modulus (*G*′) within the linear viscoelastic region followed the order D/C/M > D/C + M > D/C, indicating a progressive decrease in gel strength among the tested samples. These differences suggest that both the presence of mannitol and formulation processing history influence gel structure. The higher *G*′ of D/C/M can be attributed to a more structured hydrogen-bond network and reduced water mobility, which enhance network cohesion in hydrogels [52]. Mannitol, acting as a kosmotropic solute, enhances hydrogen bonding, strengthens hydrogel structure, and increases the fraction of less mobile water [75,76]. Kosmotropic species are known to increase the storage modulus (*G*′) of hydrogels by enhancing hydrogen bonding and promoting the formation of denser and more cohesive network structures [77]. Accordingly, mannitol, as a kosmotropic solute, may contribute to the higher elasticity observed in the D/C/M system.

These findings further support the critical role of mannitol as an excipient in the preparation of donepezil-loaded chitosan-based microspheres, highlighting that not only its presence but also formulation processing history contributes significantly to the final gel properties.

The second goal was to evaluate the effect of dehydration on the physical properties of the gel formed by D/C/M microspheres. *G*′ and *G*″ were measured as functions of shear strain (amplitude sweep) and angular frequency (frequency sweep), and apparent viscosity was determined as a function of shear rate.

Measurements were performed for the reference D/C/M gel used in the water loss studies (prepared as explained in Section 2.2.7), as well as for two more concentrated gels (D/C/M–2 h gel and D/C/M–7.5 h gel) obtained by swelling D/C/M microspheres in reduced amounts of SNF. These conditions correspond to the dehydration levels observed after 2 h and 7.5 h in water loss studies conducted using empty RC and an uncoated membrane. This setup was selected because it resulted in the highest dehydration rate of the D/C/M gel (Figure 5).

**Figure 8:**
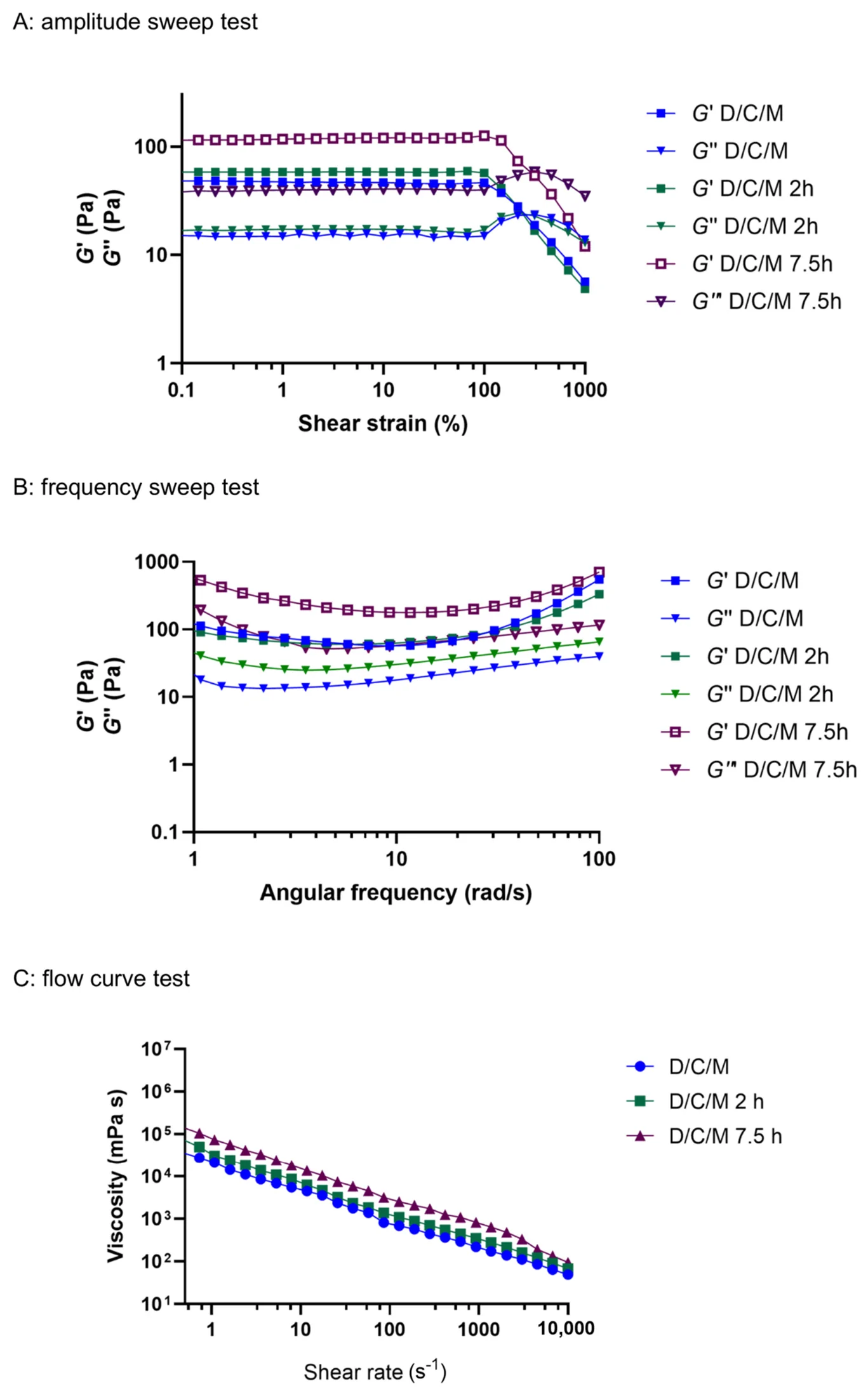
Rheological characterization (amplitude sweep test, frequency sweep test, flow curve test) of swollen D/C/M gel at dehydration time points 0 h, 2 h and 7.5 h.

The 2 h time point represents the expected residence time of powder formulations at the nasal mucosa, while the 7.5 h time point, corresponding to the final stage of the water loss study, was used as a positive control. The results are presented in Figure 8.

Amplitude sweep tests showed a minimal increase in *G*′ and *G*″ for the D/C/M–2 h gel compared to the D/C/M gel, whereas markedly higher *G*′ and G″ values were observed for the D/C/M–7.5 h gel (Figure 8A). Similarly, frequency sweep tests revealed increased G’ values for the D/C/M–7.5 h gel, while the *G*′ profiles of the D/C/M and D/C/M–2 h gels largely overlapped (Figure 8B). At a frequency of 1 Hz, *G*′ values for D/C/M, D/C/M–2 h, and D/C/M–7.5 h gels were 112.3 Pa, 117.9 Pa, and 789.5 Pa, respectively. An increase in *G*′ and *G*″ with decreasing water content in polymer-based gels has been previously reported [78].

These results indicate that the level of dehydration observed after 2 h under biorelevant temperature and humidity conditions does not substantially alter the gel’s rheological properties. This stability suggests that the powder system can be applied nasally without a risk of excessive dehydration or the formation of crusts or films during the expected residence time, which could otherwise cause irritation or reduce patient adherence. The marked increase in viscoelastic moduli observed for the D/C/M–7.5 h gel confirms the sensitivity of the method and validates its use as a positive control.

In the context of nasal drug delivery, the elastic component of mucus plays a key role in mucociliary clearance, with optimal transport reported at *G*′ values of 1–2 Pa. The *G*′ values obtained for D/C/M and D/C/M–2 h gels are substantially higher and fall within the range previously reported for metoprolol and atenolol HPC/MCC powder formulations (90.6 and 146.9 Pa, respectively) [6], indicating increased resistance to mucociliary clearance and potential for prolonged nasal residence time.

Flow curve analysis (Figure 8C) revealed a corresponding trend in apparent viscosity, with D/C/M < D/C/M–2 h < D/C/M–7.5 h, consistent with decreasing water content and mirroring the changes in *G*′. At a shear rate of 1 Hz, corresponding to the effective shear rate of ciliary beating in nasal mucus [23], these pseudoplastic systems exhibited apparent viscosities of 21,143, 27,576, and 78,154 mPa·s. Viscosity levels for D/C/M and D/C/M–2 h gels were comparable to those previously reported for 2% HPMC 4000 and 2% pectin dispersions in SNF (sieve fraction 32–90 µm) measured under the same shear rate [23]. Although some poloxamer 407 nasal gels can reach similarly high viscosities [79,80], these values are generally well above those reported for in situ gels formed from liquid formulations.

Nasal powders offer a practical alternative to liquid in situ gels, which must remain sufficiently low in viscosity to allow aerosolization and effective administration [25]. Administered dry, powders hydrate upon contact with the nasal mucosa and rapidly form a highly viscous, gel-like layer at the deposition site. This enables local viscosities that exceed those achievable with liquid systems, enhancing mucoadhesion and retention.

### 3.5. Results of Slug Mucosal Irritation Assay

In a comprehensive approach to the development of advanced nasal delivery systems, the fact that nasal powders can cause irritation and discomfort on the sensitive nasal mucosa is often overlooked [23]. Patients perceive this discomfort as sensation of stinging, itching, and burning in the nose. Identifying and minimizing the potential of nasal powders to cause such adverse effects is crucial, as it may significantly enhance patient comfort and adherence to therapy. However, these sensations are difficult to demonstrate using in vitro tests. Therefore, an in vivo slug mucosal irritation (SMI) assay was developed to predict the irritation potential of formulations, as formulation-induced mucus production was shown to correlate with irritancy in humans [24,81]. SMI assay has already been implemented in development of mucosal drug delivery systems [23,25,26].

The determination of the irritation potential of the prepared powder samples was carried out according to the protocol described in Section 2.2.9. The irritation potential of a powder formulation may be associated with the composition, size, physical state and morphology of the powder particles [13,23]. The results on the SMI assay performed with tested powder samples, as well as with positive and negative controls, are summarized in Table 5.

All tested samples differed significantly from the benzalkonium chloride (BAC, 1% *w*/*V*) solution used as a positive control (measure of severe discomfort) [23]. Exposure to D/C resulted in a significant increase in mucus production compared with phosphate-buffered saline (PBS), indicating a potential for mild irritability. The total mucus production observed for the D/C sample is consistent with results previously reported by Trenkel and Scherließ (2021) [23] where carboxymethyl chitosan exhibited high total mucus production.

Both mannitol-containing powder samples did not differ significantly from the negative control under the conditions tested, indicating a low potential to induce discomfort on the nasal mucosa.

Due to its mucoadhesive and absorption-enhancing properties, chitosan is a crucial excipient in donepezil-loaded nasal powders for nose-to-brain delivery. However, the irritation potential demonstrated in this study could limit its clinical use and negatively affect patient adherence. By incorporating mannitol as a filler/carrier, this irritation was successfully reduced, highlighting an additional advantage of this formulation strategy. SMI assay confirmed mannitol as critical excipient for improving the sensory effects of donepezil nasal powders.

## 4. Conclusions

The present study clearly demonstrates the critical role of mannitol in the formulation of swellable, spray-dried chitosan-based microspheres for nasal donepezil delivery. Mannitol was identified as a key functional component governing particle size distribution, water retention, and sensory tolerability, while maintaining process robustness. Importantly, the proposed biomimetic model provides novel insights into gel hydration, dehydration resistance, and rehydration behavior under simulated nasal conditions, offering a predictive tool for assessing mucosa-formulation water transfer and tolerability. Furthermore, the comprehensive experimental approach presented herein addresses the currently insufficiently explored prediction of nasal mucosal tolerability of swellable powder systems, thereby contributing to the rational design of safe and effective intranasal therapies.

## Figures and Tables

**Table 1:** Composition of the prepared powder samples.

Sample	D (%, *w*/*w*)	C (%, *w*/*w*)	M or L (%, *w*/*w*)
D/C/M	5	19	76
D/C/L	5	19	76
D/C + M	5	19	76
D/C	21	79	/

D—donepezil, C—chitosan, M—mannitol, L—lactose.

**Table 2:** Composition of gels in the dehydration studies, prepared by swelling the powders in 1 mL of simulated nasal fluid (SNF). The concentrations of donepezil (D), chitosan (C) and mannitol (M) are expressed as mg per mL of SNF.

Sample	D (mg mL^−1^)	C (mg mL^−1^)	M (mg mL^−1^)
D/C/M gel	5	19	76
D/C + M gel	5	19	76
D/C gel	6	23	/

**Table 3:** Experimental and control setups used in dehydration study.

Experimental Setup	Control Samples
empty RC/uncoated membrane + gel sample	empty RC/1 mL of water in sample compartment on nonpermeable membrane (control)
empty RC/mucin-coated membrane (200 µL) + gel sample	
empty RC/mucin-coated membrane (500 µL) + gel sample	
1 mL of water in RC/uncoated membrane + gel sample	1 mL of water in RC/nonpermeable Vaseline-coated membrane (control 1)
	1 mL of water in RC/SNF-presoaked noncoated membrane (control 2)
	1 mL of water in RC/no membrane (control 3)

**Table 4:** Process yield (%), particle size distribution (*D*_v_10, *D*_v_50, *D*_v_90; *n* = 5), sample volume comprising particles smaller than 10 μm (*V* < 10 µm (%)), drug loading (DL, %; *n* = 3) and entrapment efficacy (EE, %; *n* = 3) measured across three independent batches.

Preparation	Process Yield (%)	*D*_v_10 (µm)	*D*_v_50 (µm)	*D*_v_90 (µm)	*V* < 10 µm (%)	*DL* (%)	*EE* (%)
	D/C/M						
1 *	47.2	11.6 ± 0.4	29.0 ± 1.2	63.5 ± 1.1	6.0	5.1 ± 0.2	101.5 ± 2.9
2	37.3	10.4 ± 0.0	24.3 ± 0.1	55.4 ± 0.8	8.6	4.9 ± 0.1	98.0 ± 2.5
3	38.3	12.6 ± 0.3	32.0 ± 0.9	65.0 ± 0.7	6.3	5.0 ± 0.2	99.3 ± 3.5
AVG	40.9 ± 5.5	11.5 ± 1.1	28.4 ± 3.9	61.3 ± 5.2	7.0 ± 1.4	5.0 ± 0.1	99.6 ± 1.8
RSD (%)	13.3	9.6	13.7	8.4	20.2	2.4	1.8
	D/C/L						
1	43.2	5.2 ± 0.1	10.5 ± 0.1	24.1 ± 0.1	46.3	5.0 ± 0.1	99.6 ± 3.0
2	44.2	7.0 ± 0.1	13.0 ± 0.1	23.8 ± 0.5	29.4	4.6 ± 0.1	91.4 ± 2.5
3	41.5	5.0 ± 0.0	13.7 ± 0.3	32.6 ± 0.9	35.1	5.0 ± 0.0	99.1 ± 0.7
AVG	43.0 ± 1.2	5.7 ± 1.1	12.4 ± 1.7	26.8 ± 5.0	37.0 ± 8.6	4.9 ± 0.2	96.7 ± 4.6
RSD (%)	2.8	19.1	13.6	18.6	23.2	4.5	4.8
	D/C						
1 *	28.1	4.6 ± 0.0	9.7 ± 0.0	22.6 ± 0.5	52.0	20.9 ± 1.2	99.8 ± 5.8
2	29.3	6.5 ± 0.1	13.3 ± 0.2	66.8 ± 10.2	32.2	20.8 ± 0.6	99.1 ± 2.8
3	24.3	5.9 ± 0.0	11.4 ± 0.1	24.7 ± 1.2	40.2	20.2 ± 0.5	96.3 ± 2.2
AVG	27.2 ± 2.7	5.7 ± 1.0	11.5 ± 1.8	38.0 ± 24.9	41.5 ± 10.0	20.7 ± 0.4	98.4 ± 1.8
RSD (%)	9.9	17.5	15.9	65.6	24.0	1.8	1.8

AVG—average result for three independent batches; RSD—relative standard deviation. *—data previously reported in [13].

**Table 5:** Total mucus production across the three contact periods (CPs) in the slug mucosal irritation assay, expressed as a percentage of the slugs’ initial body weight.

Sample	Total Mucus Production (%)
D/C *	8.19 ± 1.79
D/C/M	5.04 ± 1.43
D/C + M	5.49 ± 2.44
PBS	0.48 ± 1.50
BAC (1%, *w*/*V*)	17.64 ± 4.33

Data are presented as mean ± SD (*n* = 3). *—statistically significant difference in TMP with respect to PBS, *p* < 0.05.

## Data Availability

The original contributions presented in this study are included in the article/Supplementary Material. Further inquiries can be directed to the corresponding author.

## Supplementary Materials

The following supporting information can be downloaded at https://www.mdpi.com/article/10.3390/pharmaceutics18081023/s1, Figure S1. DSC thermogram (A) and XRPD diffractogram (B) of the D/C/M sample stored at 4 °C during a 4-year period (D/C/M—2022) and a freshly prepared one (D/C/M—2026).

## Abbreviations

The following abbreviations are used in this manuscript:

BAC	benzalkonium chloride
BW	body weight
CP	contact period
DL	drug loading
DSC	differential scanning calorimetry
EE	entrapment efficacy
HPLC	high-performance liquid chromatography
PBS	phosphate-buffered saline
RC	reservoir compartment
SMI	slug mucosal irritation assay
SNF	simulated nasal fluid
TMP	total mucus production
WER	water evaporation loss
XRPD	X-ray powder diffraction
