# Supplementary material for: Mannitol as a Critical Excipient in Spray-Dried Chitosan Microspheres for Nasal Donepezil Delivery: Insights from Integrated Biomimetic Models
Source: Pharmaceutics. 2026 Aug 18;18(8):1023. doi: 10.3390/pharmaceutics18081023 (PMC13516387; doi:10.3390/pharmaceutics18081023)
Supplement: Supplementary file 1 [file pharmaceutics-18-01023-s001.zip › pharmaceutics-4373266-supplementary.pdf]

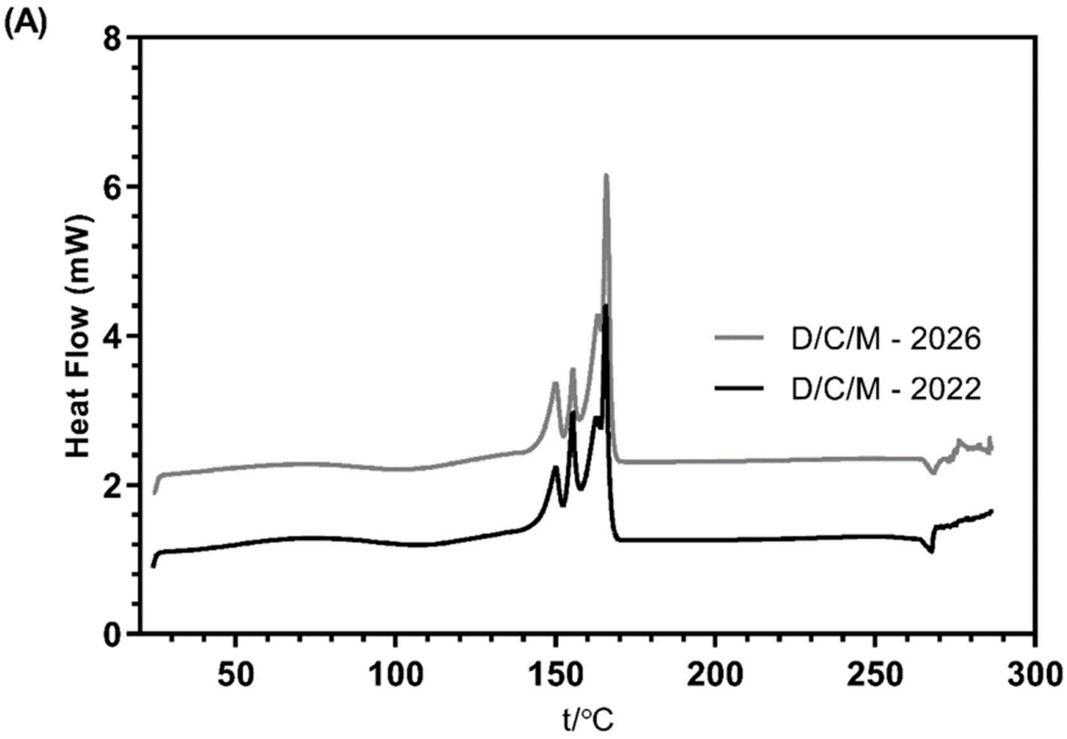


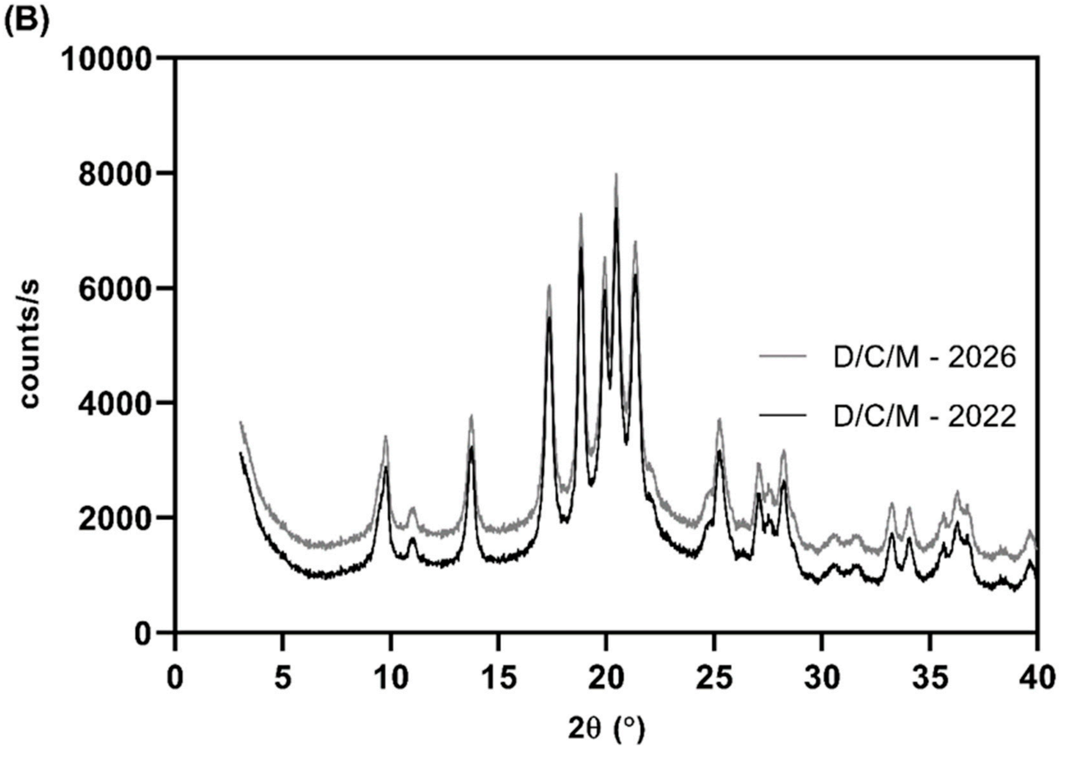


**Figure S1.** DSC thermogram (A) and XRPD diffractogram (B) of the D/C/M sample stored at 4 °C during a 4-year period (D/C/M – 2022) and a freshly prepared one (D/C/M – 2026).
